# Let’s Work Together — Occupational Factors and Their Correlates to Prison Climate and Inmates’ Attitudes Towards Treatment

**DOI:** 10.3389/fpsyt.2019.00781

**Published:** 2019-10-29

**Authors:** Julia Sauter, Joanna Vogel, Katharina Seewald, Joscha Hausam, Klaus-Peter Dahle

**Affiliations:** ^1^Institute of Forensic Psychiatry, Charité – Universitätsmedizin Berlin, Berlin, Germany; ^2^Research & Development Division, Berlin Prison and Probation Services, Berlin, Germany; ^3^Institute of Psychology, University of Hildesheim, Hildesheim, Germany

**Keywords:** occupational factors, prison staff, job satisfaction, team climate, prison climate, sick days, offender treatment, treatment motivation

## Abstract

The role of psychosocial and structural occupational factors in mental health service provision has broadly been researched. However, less is known about the influence of employees’ occupational factors on inmates in correctional treatment settings that mostly seek to apply a milieu-therapeutic approach. Therefore, the present study investigated the relationships between occupational factors (job satisfaction, self-efficacy, and the functionality of the organizational structure) and prison climate, the number of staff members’ sick days as well as inmates’ treatment motivation. Employees (*n* = 76) of three different correctional treatment units in Berlin, Germany, rated several occupational factors as well as prison climate. At the same time, treatment motivation of *n* = 232 inmates was assessed. Results showed that higher ratings of prison climate were associated with higher levels of team climate, job satisfaction and the functionality of the organizational structure, but not with self-efficacy and sick days. There was no significant relationship between occupational factors and the perceived safety on the treatment unit. Inmates’ treatment motivation was correlated with all aggregated occupational factors and with average sick days of staff members. Outcomes of this study strongly emphasize the importance of a positive social climate in correctional treatment units for occupational factors of prison staff but also positive treatment outcomes for inmates. Also, in the light of these results, consequences for daily work routine and organizational structure of prisons are discussed.

## Introduction

In prisons, genuinely multi-disciplinary and challenging working environments, occupational factors have only been given scarce scientific attention in the last decades. Studies on this subject reported low levels of job satisfaction to have a significant effect on negative work outcomes such as reduced work inclusion (1), turnover intent and actual staff turnover (2–6) and also on absenteeism (7–9). More recently, depersonalization which is one of the indicators of burnout syndrome, has been linked with increased turnover intent and absenteeism among prison staff (10). Thus, job strain that is perceived to be impossible to cope with increases the risk of certain psychological burden syndromes such as burnout (10).

In their meta-analysis, Dowden and Tellier (11) examined the predictors of work-related stress in correctional officers. The authors demonstrated that perceived safety of the workplace and role difficulties, as well as attitudes to work, were strongly related to stress. Also, both positive and punitive attitudes towards inmates were moderately related to stress at work. Nonetheless, Waters (12) found that positive correctional work settings that underline involvement, coworker cohesion and administrative support, could have supportive effects and reduce correctional officers experienced stress levels. In this respect, self-efficacy as the personal judgement of “how well one can execute courses of action required to deal with prospective situations” (13) plays an important role. Promising findings on self-efficacy and health outcomes in prison staff reveal that high levels of dispositional optimism, self-esteem, self-efficacy and perceived social support significantly enhance health in prison staff (14).

In any case, absenteeism and staff turnover are additionally financially expensive for correctional organizations (15). Furthermore, as missing staff is enlarging the staff–inmate ratio, the quality of professional staff–inmate relationships and of social climate on prison wards can hardly remain unaffected.

Very little is known about the relationship between social climate in prison and occupational factors. To our knowledge, only two studies have investigated this relationship. Moos and Schaefer (16) found improved job performance to be associated with positive climate ratings. Røssberg and Friis (17) found that positive ratings of climate were associated with higher ratings of staff satisfaction.

### Occupational Factors and Their Impact on Offender Treatment

Only few studies have investigated the impact of occupational factors on inmates in correctional settings. Most of the existing studies focused on job satisfaction as an occupational factor. Thus, job satisfaction was associated with inmates perceiving less danger of sexual assault (18), a higher support for a human-service orientation among correctional security staff (19), and a more positive view of inmates and an affirmative attitude towards rehabilitation (20). Similarly, job satisfaction was also found to be negatively associated with a punitive orientation towards inmates (21). In general, the literature suggests that higher job satisfaction of staff is related to positive work outcomes which could benefit both staff and inmates through better staff–inmate relations (22), as well as improving correctional standards and conditions (23).

However, to our knowledge, no study has yet addressed the impact of further occupational factors (e.g. team climate, self-efficacy or the functionality of the organizational structure) on inmate related work outcomes. However, in organizational psychology literature team climate has been linked to many positive work outcomes such as superior clinical care in diabetes, more positive patient evaluations of practice and self-reported innovation and effectiveness (24; for a review on health care team effectiveness and its link to team climate see 25). Also, self-efficacy has been given more and more attention as it has been found to be associated with positive work outcomes as well (for a meta-analysis on self-efficacy and work-related performance see 26).

### Research Aims and Hypotheses

This research focuses on how occupational factors among prison employees are associated with prison climate and inmates’ attitudes towards correctional treatment. It was hypothesized that staff members’ ratings on prison climate are correlated with occupational factors such as team climate, job satisfaction, self-efficacy and the functionality of the organizational structure. Furthermore, it was assumed that occupational factors are correlated with the number of staff members’ sick days. Finally, it was presumed that occupational factors as well as correctional officers’ number of sick days are correlated with inmates’ attitudes towards treatment, and thus also with the effectiveness of treatment.

## Methods

### Study Design and Procedure

On behalf of the Senate Administration for Justice and Consumer Protection, the Institute for Forensic Psychiatry of the Charité evaluates all treatment facilities for persons, who committed offences, located in Berlin prisons. The current study is part of this on-going evaluation.

According to the research question, all three treatment facilities for male individuals were included in the study. Prison-based treatment in Germany is predominantly provided in social-therapeutic facilities. The majority of these, including those located in the state of Berlin, follow a milieu-therapeutic approach (27). Another treatment facility required by law is to be found in the area of high-risk offenders. So-called preventive detention refers to a (potentially infinite) confinement practice of a selection of very high-risk sexual and violent offenders following a multi-year prison sentence and previous convictions. After several legislative changes, preventive detention is now focusing on psychosocial therapeutic treatment and support, which are similar to the treatment programs in social-therapeutic facilities. The preventive detention unit, the male as well as the adolescent social-therapeutic facilities in Berlin are located in separate units within prison. Thus, inmates can use the infrastructure (e.g. work and school) of the prison.

Data was collected between 2014 and 2016. The survey of staff members took place in semi-structured interviews, which lasted between one and a half and two hours and included open questions (not part of this study) as well as various questionnaires. Participation was voluntary; staff members who agreed to participate gave written informed consent. All psychologists/social workers of the facilities were asked to participate (participation rate: 54.2%). As for correctional officers, in a first step for economic reasons one third was randomly selected to be invited to participate in the interview in the social-therapeutic facility for male adult offenders and in the social-therapeutic facility for male adolescents. One hundred percent of those who agreed to take part and an additional number of *n* = 12 volunteered to be interviewed. In the preventive detention unit, a quota sample was taken by computer-aided random sampling, taking gender into account (female: 29.6%), and including 50% (*n* = 27) of the correctional officers. This resulted in an overall participation rate of rate: 47.0%.

The aim of the evaluation was to complete a full survey of all persons treated in a facility of the Berlin prisons. Thus, all inmates that were present at the facilities between 2014 and 2016 were asked to participate in a semi-structured interview, which lasted between two and three hours and included open questions (not part of this study) as well as various questionnaires. Participation was voluntary. After detailed information on the aim and procedure of the study by a member of the research group, persons who agreed to participate gave written informed consent. Each participant received a financial compensation of 15€. The overall participation rate was 86.6% (social-therapeutic facility for adult male offenders: 88.0%, *n* = 125 of 142; social-therapeutic facility for adolescent offenders: 96.1%, *n* = 74 of 77; preventive detention unit: 78.6%, *n* = 33 of 42).

### Participants

The overall staff sample consisted of 63 correctional officers and 13 psychologists/social workers (*n* = 76). Specifically, the sample was composed of 20 correctional officers and seven psychologists/social workers of the social-therapeutic facility for adult male offenders (age: *M* = 49.4 years; *SD* = 8.23; *Min–Max* = 34–59), 16 correctional officers and four psychologists/social workers of the social-therapeutic facility for adolescent offenders (age: *M* = 47.7 years; *SD* = 7.80; *Min–Max* = 37–59) and 27 correctional officers and two psychologists of the preventive detention unit (age *M* = 46.4 years; *SD* = 8.94; *Min–Max* = 30–57).

The overall offender sample consisted of *n* = 232 individuals. Largest subsamples were collected from the social-therapeutic facility for male adult offenders (*n* = 125; age: *M* = 41.4 years; *SD* = 10.8; *Min–Max* = 22–67; treatment duration: *M* = 24.7 months; *SD* = 24.3; *Min–Max* = 0-142; prior criminal record = 73.9%) and male adolescent offenders (*n* = 74; age: *M* = 19.9 years; *SD* = 1.8; *Min–Max* = 16–23; treatment duration: *M* = 11.8 months; *SD* = 10.6; *Min–Max* = 0–47; prior criminal record = 98.2%), whereas a smaller group was collected from the facility for offenders under preventive detention (*n* = 33; age: *M* = 40.1 years; *SD* = 9.7; *Min–Max* = 36–74; treatment duration: *M* = 58.4 months; *SD* = 36.9; *Min–Max* = 6–179; prior criminal record = 100%). Convicted for violent offenses (homicide, aggravated assault, battery, and robbery) were *n* = 111 persons (47.8%), 57 persons (24.6%) for rape, 53 persons (22.7%) for sexual abuse of children, and 11 persons (4.8%) for other offenses such as aggravated theft. Persons with a concurrent conviction for a sexual and a violent offense were categorized as sexual offenders.

### Measures

Except for the measure to assess prison climate, the questionnaires used have been designed and validated for other occupational groups (e.g., nurses). Therefore, it was partly necessary to adapt and reformulate items for the prison context. In addition, the questionnaires were partially shortened to save time and resources. The selection of the items was based on empirical (e.g., highest factor loadings) and conceptual considerations (e.g., appropriateness for prison context). The questionnaires are available upon request.

#### Staff Members’ Ratings

##### Prison Climate

The perception of prison climate was measured by the Essen Climate Evaluation Scheme – Prison Version (EssenCES; 28). The questionnaire entails 17 items that are answered on a 5-point Likert scale, from 1 (strongly disagree) to 5 (strongly agree). Higher scores indicate more favorable levels of prison climate. Except for two filler items, the 15 items can be divided into three subscales with five items each: a) *therapeutic hold*, b) *inmates’ cohesion*, and c) *perceived safety*. The EssenCES has robust psychometrics (Cronbach’s α ranged from .76 to .85 in theGerman norm sample; 29) and showed meaningful associations with a positive working environment, institutional aggression, and site security (29).

##### Team Climate

The Team Climate Inventory (TCI; 30, 31) is a psychometric questionnaire for measuring work atmosphere in groups. The questionnaire consists of 44 items that are assigned to four subscales (*vision, task orientation, safety, and support for innovation*) and a check scale for socially desirable response behavior. The items are answered on a 5-point scale, from 1 (strongly disagree) to 5 (strongly agree). For the present study, the TCI was shortened down to 15 items, which cover three subscales (1) *safety* (5 items), (2) *vision* (7 items), and (3) *task orientation* (3 items). The total score indicates an overall level of team climate, with higher ratings referring to a better team climate as perceived by staff. Previous research on the TCI suggested that higher levels of team climate were associated with reduced intentions to leave and turnover in hospital staff (32). The shortened version of the questionnaire has shown excellent internal consistency in the current sample as indicated by by Cronbach’s α = 0.93.

##### Job Satisfaction

To gather data on job satisfaction an unpublished adaption derived from the Job Descriptive Index (JDI; 33) and the SAZ (Skala zur Erfassung der Arbeitszufriedenheit; 34, 35) was used. The developed *job satisfaction* scale entails 8 items concerning satisfaction with colleagues, supervisor, work task, working conditions, organization, management, work load and opportunities. The items are answered on a 5-point-Likert scale, from 1 (completely unsatisfied) to 5 (completely satisfied). Higher scores indicate higher levels of job satisfaction. The adapted version of the questionnaire has shown good internal consistency as indicated by Cronbach’s α = .85.

##### Self-Efficacy

Two unpublished versions (for teachers and nursing staff) of the general self-efficacy scale (SWE; 36) were adopted for use in social-therapeutic facilities. The 5 items of the questionnaire assess self-efficacy of staff in dealing with difficult and suspicious inmates. Each item is answered on a 4-point scale, from 1 (strongly disagree) to 4 (strongly agree). The total score indicates an individual level of self-efficacy. Higher scores indicate higher levels of perceived self-efficacy. The adapted version of the questionnaire has shown poor internal consistency in the current sample as indicated by Cronbach’s α = .58. Thus, it is still above the threshold for rejection (Cronbach’s α < .50; 37) and has demonstrated a high level of content validity assessed by expert opinion.

##### Functionality of the Organizational Structure

Since no suitable questionnaire for recording the functionality of the organizational structure was available prior to this project survey, the main points in this area were extracted from interviews with practitioners and experts in the field. To assess relevant aspects of the organizational structure, staff members were asked to grade eight aspects of their organization and working team: (1) professionalism, (2) specialist qualification, (3) commitment and motivation, (4) cooperation between officers and psychologists/social workers, (5) recognition of the officers work by psychologists/social workers or vice versa, (6) respect for the work of the treatment unit by the staff of regular prison units, (7) support and encouragement by the management within the treatment unit, (8) flow of information by the management of the treatment unit. Grades ranged from 1 (very good) to 5 (insufficient), an average grade was calculated. For better illustration, the eight questions were mapped in the form of a target, with the best grade in the middle and the worst at the edge of the target. The newly developed questionnaire has shown acceptable internal consistency as indicated by Cronbach’s α = .76.

##### Sick Days

The number of days of absence due to sickness per employee per year (*sick days*) were anonymously obtained by the head office.

#### Offenders’ Ratings

##### Attitudes Towards Treatment

Negative attitudes towards treatment were measured by the Therapiebezogene Einstellungen [attitudes towards treatment]-Short-Version (TBE-SV; 38; 39). The TBE-SV consists of 24 items, which can be divided into 5 subscales. Each item is answered on a 4-point Likert scale, from 1 (I do not agree) to 4 (I do agree). The first subscale, (I) *trust in therapy*, measures general beliefs about the helpfulness of treatment. This is the only subscale with positive encoding, i.e. higher scores indicate a positive attitude. The four remaining scales are reverse coded, i.e. higher scores indicate negative attitudes towards treatment. (II) *Mistrust in mental health professionals* measures resentments and suspicions about therapeutic staff. (III) *Therapy restraint* measures the level of aversive attitudes towards one’s own treatment. (IV) *Fear of stigmatization* measures feelings of shame about looking for help from therapeutic staff, and (V) *fear of self-disclosure* measures hesitations about opening up to others in a therapeutic context. A total score can be computed with the formula (*30 – subscale I*) *+* (*II+III+IV+V*) and indicates an overall level of *therapy resistance* (high values indicate high resistance against therapy). Dahle (39) reported acceptable internal consistencies as indicated by Cronbach’s α, ranging from .68 to .81 for the subscales. The readiness to enter a subsequent treatment offer could be predicted by the total score (39). Offenders’ *therapy resistance* was negatively associated with perceived prison climate, *r* = −.28 (40).

### Statistical Analyses

Statistical analyses were performed with SPSS 20.0 for Windows (41). Bonferroni-corrections were applied to all tests. Due to the exploratory character of the study and no clearly directed hypotheses, correlation analyses have been favored to assess the relationship between the variables in question. Pearson Correlations were calculated between the individual occupational factors and individual prison climate ratings from staff members. Pearson Correlations were also calculated for the occupational factors and sick days. Next, data from staff members’ ratings (but not offenders’ ratings) were aggregated by calculating means individually for each treatment unit. These means were then transferred to offenders’ rating by assigning means from one facility to offenders’ ratings from that specific treatment unit. This operation makes it possible to correlate means of staff members’ ratings with individual offenders’ ratings on prison climate as well as their therapy resistance measured by the TBE-SV. Theoretically this is based on Kozlowski and Klein (42) who stated that “a phenomenon is emergent when it originated in the cognition, affect, behaviours, or other characteristic of individuals, is amplified by their interactions, and manifests as a higher-level, collective phenomenon.” According to them, through the process of emergence originally individual phenomena become so-called “shared unit properties” which are identified as properties of an organization. Occupational factors in our study can be seen as these so-called shared unit properties. Thus, aggregation of the data can be justified on a statistical level: Aggregation of data is verified if members of a group are consistent in their perceptions of a phenomenon (43). Therefore, intraclass-correlations (ICC) were calculated to check for consistency of the data. There is no strict standard on ICC-values that verify aggregation of data (44). However, values above an ICC ≥ .60 indicate good agreement in clinical settings and was therefore set as cut-off (45). All occupational factors satisfied this criterion, ICCs were as follows: Team Climate (ICC = .91), Job Satisfaction (ICC = .82), Self-Efficacy (ICC = .61), F-O-Structure (ICC = .78). Aggregation is verified, if the F-value in a variance analysis is statistically significant, which means that the variance between the groups is bigger than within the group (46). For all occupational factors this was the case except for self-efficacy. A possible explanation was that self-efficacy refers to individual rather than group-targeted construct. However, as indicated above Self-Efficacy still has a good ICC, which is why we decided to include it for further calculations (42, 45). Descriptions of staff members’ ratings are displayed in **Table 1** individually for each treatment unit. Lastly, Pearson’s Correlations were calculated between aggregated occupational factors and individual offenders’ ratings, as well as between individual staff members’ ratings. Data were normally distributed as indicated by the Shapiro-Wilk test (results ranging from *W* = .97 to .95; *p* > .05); therefore Pearson’s Correlations are justified. Missing data were dealt with by pairwise deletion. For all tests, alpha level was set at *p* < .05.

**Table 1:** Psychometric Measures for Staff Members (n = 76) and Inmates (n = 232).

	*M*	*SD*	*Min*	*Max*
**Staff members’ rating from social-therapeutic treatment unit for adults (n=27)**				
*Team Climate*	56.41	9.56	37.00	75.00
*Job Satisfaction*	26.26	5.80	14.00	36.00
*Self-Efficacy*	15.48	1.83	13.00	20.00
*F-O-Structure*	22.80	4.86	13.00	31.00
*Sick Days*	50.70	–	–	–
**Staff members’ rating from social-therapeutic treatment unit for adolescents (n=20)**				
*Team Climate*	52.25	6.57	41.00	64.00
*Job Satisfaction*	25.40	4.02	19.00	33.00
*Self-Efficacy*	14.75	1.80	11.00	18.00
*F-O-Structure*	23.79	4.74	16.00	30.00
*Sick Days*	69.65	–	–	–
**Staff members’ rating from treatment unit for inmates under preventive detention (n=29)**				
*Team Climate*	57.39	7.60	38.00	67.00
*Job Satisfaction*	29.79	4.48	14.00	35.00
*Self-Efficacy*	14.17	2.02	11.00	20.00
*F-O-Structure*	23.29	4.85	16.00	36.00
*Sick Days*	23.50	–	–	–
**Inmates from all facilities (n=232)**				
*SUM: therapy resistance*	60.15	11.46	33.00	97.25
*(1) Trust in Therapy*	15.74	2.91	5.00	20.00
*(2) Mistrust in Mental Health Professionals*	11.19	3.21	5.00	20.00
*(3) Restraint to Therapy*	11.18	3.25	5.00	20.00
*(4) Fear of Stigmatization*	11.47	2.47	5.00	18.00
*(5) Fear of Disclosure*	12.04	3.34	5.00	20.00

F-O-Structure, Functionality of the organizational structure.

## Results

### Occupational Factors and Their Correlations to Prison Climate

*Team climate* and the *functionality of the organizational structure* correlated significantly with the subscales *therapeutic hold* and *inmates’ cohesion* of the EssenCES with correlation coefficients ranging from .31 to .52. *Job satisfaction* correlated positively with *inmates’ cohesion* (*r*(74) *=* .24, *p* < .05), *self-efficacy* with *therapeutic hold* (*r*(74) = .34, *p* < .01). There was no correlation between occupational factors and the subscale *perceived safety* of the EssenCES, neither was there a correlation between *sick days* and overall *prison climate* ratings according the EssenCES. Results are fully presented in **Tables 1** and **2**.

**Table 2:** Pearson Correlations between Occupational Factors as well as the number of sick days from Staff Members and their Prison Climate ratings *(n* = 76*)*.

Occupational Factors	Prison Climate measured by the EssenCES				Number of Sick Days
***Sum***	***Perceived Safety***	***Therapeutic Hold***	***Inmates’ Cohesion***
*Team Climate*	.53**	.20	.43**	.52**	–.17
*Job Satisfaction*	.23*	.07	.19	.24*	–.36***
*Self-Efficacy*	.13	–.13	.34**	.17	–.04
*F-O-Structure*	–.37**	–.16	–.33**	–.31**	–.16
*Sick Days*	–.05	–.01	.04	–.16	1

F-O-Structure, Functionality of the organizational structure. * p < .05, ** p < .01, *** p < .001.

### Occupational Factors and Their Correlations to the Number of Sick Days

No correlations were found between *team climate*, *self-efficacy*, *functionality of the organizational structure* and *sick days*. However, a correlation of *r*(74) = −.36, *p* < .001) was found between *sick days* and *job satisfaction* (see **Table 2**).

### Occupational Factors and Their Correlations to Inmates’ Attitudes Towards Treatment

Bivariate correlations were also calculated for aggregated *occupational factors*, *sick days* and individual *therapy resistance (attitudes towards treatment)* ratings from inmates (see **Tables 1**and **3**). The results are quite similar to the staff members’ ratings. *Team climate* and the *functionality of the organizational structure* correlated significantly with all subscales and consequently with the sum of the *therapy resistance* questionnaire completed by inmates, demonstrating that positive *team climate* was associated with lower *therapy resistance* (notice: subscale (I) is reversed poled: higher ratings indicate a higher trust in therapy). The same applies to the *functionality of the organizational structure*. A poor marking by staff members is related to higher *therapy resistance* on inmates’ side. *Job satisfaction* and *self-efficacy* show a similar trend even though a significant correlation was not found in all subscales. Thus, *job satisfaction* correlated with *fear of stigmatization* (*r*(230) *=* −.14, *p* < .05) and *fear of disclosure* (*r*(230) *=* −.15, *p* < .05) and *self-efficacy* with *mistrust in therapists* (*r*(230) *=* −.15, *p* < .05) and *fear of disclosure* (*r*(230) = −.18, *p* < .01).

**Table 3:** Pearson Correlations between aggregated Occupational Factors and Sick Days with individual Attitudes Towards Treatment ratings from Inmates *(n* = 232*)*.

Occupational Factors	Attitudes towards treatment (TBE-SV)					
	*SUM*	*(I)*	*(II)*	*(III)*	*(IV)*	*(V)*
*Team Climate*	–.33**	.17*	–.24**	–.26**	–.24**	–.32**
*Job Satisfaction*	–.17*	.12	–.12	–.12	–.14*	–.15*
*Self-Efficacy*	–.16*	.04	–.13	–.15*	–.10	–.18**
*F-O-Structure*	.33**	–.16*	.24**	.27**	.24**	.33**
*Sick Days*	.26**	–.15*	.18**	.19**	.19**	.24**

F-O-Structure, Functionality of the organizational structure. *p < .05, **p < .01, ***p < .001.

SUM = therapy resistance: (I) trust in therapy; (II) therapy restraint; (III) mistrust in therapists; (IV) fear of stigmatisation; (V) fear of self-disclosure.

Quite different from staff members’ perceived *prison climate* there was a highly significant correlation between the number of sick days from staff members and inmates’ quoted *therapy resistance* (r(230) = .26, p < .01). A high number of sick days on staff members’ side was connected to all of the subscales of *therapy resistance* (see **Table 3**).

## Discussion

The study aimed at investigating the impact of occupational factors on prison climate and inmates’ attitudes towards treatment. Reports of a good team climate and a functional organizational structure correlated significantly with perceived therapeutic hold and inmates’ cohesion which indicates a connection between system variables and positive treatment factors. Accordingly, measures to improve team climate and working relationships are not only an investment in the organizational culture itself but also indirectly in therapeutic factors, and insofar have an impact on inmates as well. In other words, a positive intra- and inter-group climate could function as a crucial resource for correctional settings as a milieu-therapeutic community (e.g. exemplary function). Moreover, the organizational structure seems to play an important role: Feeling adequately informed and valued by the leaders, feeling well qualified for their work, receiving regular training that meets the needs as well as a respectful cross-professional collaboration, was positively related to prison climate on treatment units.

In accordance with the aforementioned findings, personal job satisfaction correlated with inmates’ cohesion. Previous studies showed that job satisfaction was related to a more positive view of inmates (20), a less punitive orientation towards inmates (21) and inmates perceiving less danger of sexual assault (18). Different from previous findings, there was no connection with the therapeutic hold (20). However, self-efficacy was linked to therapeutic hold.

An intriguing result is that none of the occupational factors correlated with the perceived safety on prison units. An explanation could be that persons who are willing to work in a prison are aware of potential risks. When completing the questions about their perceived safety, this could lead to a lower overall level of sensitivity. It is also conceivable, however, that for the same reason for this group of people, safety aspects might not influence work-related questions.

Surprisingly, there was no significant connection between the number of staff members’ sick days and their perception of prison climate on the units at all. Nevertheless, the number of sick days negatively correlated highly with staff members’ job satisfaction in general. Perhaps this may indicate that a retreat in sick days in uncomfortable situations at work is more contingent on personality traits and/or experienced stress (7, 11) than on the general climate on the prison unit. Thus, recruitment procedures should take that into account, especially because staff turnover is very costly for correctional organizations (15). Hence, further investigation of the reasons for absenteeism in prison is warranted.

As presumed, a positive team climate and a functional organizational structure as described above were not only positively connected with prison climate but also with inmates’ attitudes towards treatment. When prison staff feel adequately informed and valued by the leadership, well qualified for their work, trained regularly according to their needs, and respected within the cross-professional collaboration, inmates seem to be able to trust in therapy and show less therapy restraint. Similar to the above findings, job satisfaction was connected with the atmosphere on the unit. Thus, there was no correlation with therapeutic variables but with the fear of being stigmatized and personal information being disclosed by others. Likewise, staffs self-efficacy as a personal construct was connected with inmates’ lower restraint to therapy and fear of disclosure but not with inmates’ general beliefs in therapy. It is assumable that self-efficacy of staff delivering treatment influences a specific therapeutic relationship but not an overall attitude towards treatment. Further research is needed to investigate these differences. Nevertheless, overall ratings of therapy resistance showed significant correlations with both job satisfaction and self-efficacy.

A result of particular importance was the overall correlation between the number of staff members’ sick days and inmates’ attitudes towards treatment. A higher number was connected with less trust in therapy, higher mistrust in mental health professionals, higher restraint to therapy, higher fear of stigmatization, and disclosure. A high number of sick days causes mistrust, which might lead to a treatment attrition (47). It can be assumed that inmates feel left behind and surrendered. While team climate, self-efficacy, and the functionality of the organizational structure seem not to be associated with sick days, first indications of sick days being caused by job dissatisfaction could be identified. It has already been shown that job absenteeism can be caused by job stress (7). In turn, job stress is caused by perceived dangerousness, role difficulties, and favorable as much as unfavorable attitudes towards inmates (11). Therefore, by realizing a milieu-therapeutic approach it might be advisable to take staff members’ attitudes towards inmates into account. A fundament for this could be team and case supervision on a regular basis to build awareness of staff members’ favorable and unfavorable attitudes towards inmates.

There are several limitations in the current study. The self-efficacy questionnaire, which was adapted to the prison context, has shown questionable internal consistency. Therefore, results involving the self-efficacy questionnaire have to be interpreted very carefully. In future studies the adaption of the original scale has to be further investigated in order to improve psychometric measures. Since it was a partial analysis of an on-going evaluation project of correctional facilities, most of the participants in this study are still in detention. Thus, we could not test the long-term effect of occupational factors and inmates’ attitudes towards treatment on long-term outcomes such as recidivism. The sample itself is relatively heterogeneous regarding their age and offense types since they are from three different correctional facilities. Also, larger sample sizes and non-selective staff samples need to be investigated in order to confirm the results. Most of all the correlations can only show an interdependence between variables. Longitudinal studies are needed to investigate the direction of the linear correlations. Only then, more elaborate practical implementations can be deducted from the results.

Nonetheless, the present study could show that occupational factors can be linked to prison climate and inmates’ attitudes towards treatment. Therefore, creating a good working environment may not only support the employees of a prison but also create a constructive therapeutic setting, which can provide a continuous support for persons in detention. The study was an attempt to contribute to these clearly under-researched issues. The results indicated that occupational factors need further investigations not only for the sake of the prison staff but also for the sake of treatment outcomes. Future research should focus more on establishing programs to promote a positive team climate, increased job satisfaction, and self-efficacy. Furthermore, the hierarchical structure of prisons and its effects on working variables should be addressed in future research when investigating treatment outcomes.

## Ethics Statement

This study was carried out in accordance with the recommendations of the Senate for Justice, Consumer Protection and Anti-Discrimination of Berlin, Germany with written informed consent from all subjects. All subjects gave written informed consent in accordance with the Declaration of Helsinki. The protocol was approved by the Official Data Protection Officer of Charité - Universitätsmedizin Berlin. Ethical approval for the study was sought and granted by the Ethics Committee of Charité - Universitätsmedizin Berlin (EA4/131/18).

## Author Contributions

JS and JV conceived of the present idea, developed the theory, performed the computations, and wrote the first draft of the manuscript. KS wrote sections of the manuscript. KS, JH, and K-PD verified the analytical methods. K-PD supervised the project. All authors discussed the results and contributed to the final manuscript.

## Funding

The author(s) disclosed receipt of the following financial support for the research, authorship, and/or publication of this article: The Senate of Justice and Consumer Protection of Berlin, Germany funded the evaluation project.

## Disclaimer

The views expressed are those of the authors and not necessarily those of the Senate of Justice and Consumer Protection of Berlin.

## Conflict of Interest

The authors declare that the research was conducted in the absence of any commercial or financial relationships that could be construed as a potential conflict of interest.
